# Predictors of Physical Resilience in Older Adults

**DOI:** 10.1093/geroni/igaa057.928

**Published:** 2020-12-16

**Authors:** Catherine O’Brien, Roee Holtzer

**Affiliations:** Yeshiva University, Bronx, New York, United States

## Abstract

Physical resilience (PR), which denotes one’s ability to resist functional physical decline, can be operationalized through longitudinal assessments on the Short Physical Performance Battery (SPPB). Dual-task walking (DTW) is predictive of adverse outcomes but its role in predicting incident PR has not been assessed. Herein, we determined whether velocity during Single-Task-Walk (STW) and Dual-Task-Walk (DTW) conditions predicted incident loss of PR and identified moderators of this relationship. Participants were 163 (mean age=75.5; %female=52) non-demented, community-dwelling older adults with baseline SPPB scores of 10-12. At baseline, individuals completed neuropsychological testing, the SPPB and DTW paradigm. Cognitive reserve was evaluated using the Wide Range Achievement Test (WRAT-3) and speed of processing was assessed using the Symbol Digit Modalities Test (SDMT). Individuals with SPPB scores < 10 were categorized as not physically resilient. Those with scores of 10 or higher were categorized as physically resilient. At three-year follow up 75.4% (n=123) of participants remained physically resilient while 24.5% (n=40) lost PR. Binary logistic regression revealed that slower DTW (OR= 0.96, p= 0.033, 95%CI [.926, .997]), but not STW velocity (OR= 1.00, p= 0.861, 95%CI [0.962, 1.048]), was a significant predictor of PR loss. Moreover, moderation analyses revealed that DTW velocity predicted PR loss only among individuals who had lower baseline scores on the WRAT-3 (OR=0.937, p=0.004, 95%CI [.896, .979]) and SDMT (OR=0.949, p=0.018, 95%CI [.909, .991]). We propose that cognitive reserve and speed of processing influenced the utility of DTW velocity in predicting PR loss among community-residing older adults.

